# Predictors of atrial fibrillation in ibrutinib-treated CLL patients: a prospective study

**DOI:** 10.1186/s13045-018-0626-0

**Published:** 2018-06-11

**Authors:** Gianluigi Reda, Bruno Fattizzo, Ramona Cassin, Veronica Mattiello, Tatiana Tonella, Diana Giannarelli, Ferdinando Massari, Agostino Cortelezzi

**Affiliations:** 1 UOC Ematologia, Fondazione IRCCS Ca’ Granda Ospedale Maggiore Policlinico di Milano, via Francesco Sforza 35, 20135 Milan, Italy; 2 UOC Ematologia, Fondazione IRCCS Ca’ Granda Ospedale Maggiore Policlinico di Milano, Università degli Studi di Milano, via Francesco Sforza 35, 20135 Milan, Italy; 3 UOC Malattie Cardiovascolari, Fondazione IRCCS Ca’ Granda Ospedale Maggiore Policlinico di Milano, Università degli Studi di Milano, via Francesco Sforza 35, 20135 Milan, Italy; 4 Bio-statistical Unit, Regina Elena National Cancer Institute, via Elio Chianesi 53, 00144 Roma, Italy

**Keywords:** Chronic lymphocytic leukaemia, Ibrutinib, Atrial fibrillation, Cardio-oncology

## Abstract

**Background:**

Ibrutinib is an oral irreversible inhibitor of Bruton’s tyrosine kinase, indicated for the treatment of chronic lymphocytic leukaemia. The drug is generally well tolerated; however, not infrequent side effects are reported, with the major two being bleeding and ibrutinib-related atrial fibrillation. Atrial fibrillation pathogenesis in this setting is not completely clear, and no prospective studies have evaluated the impact of previous cardiologic history and baseline characteristics.

**Methods:**

We prospectively performed cardiologic assessment in 43 CLL patients before starting ibrutinib therapy. Cardiologic workup included comorbidity collection and electrocardiographic and echocardiographic baseline evaluation.

**Results:**

After a median observation of 8 months, seven patients developed atrial fibrillation (16.3%). Cases developing atrial fibrillation were all elderly males (*p* = 0.04), and mostly with a history of previous arterial hypertension (*p* = 0.009). Atrial fibrillation occurrence also correlated with the presence of one or more pre-existent cardiologic comorbidities (*p* = 0.03), with a higher atrial fibrillation risk score (calculated with comorbidities and cardiologic risk factor evaluation *p* < 0.001), and with higher left atrial diameter (*p* = 0.02) and area (*p* = 0.03) by echocardiography. The occurrence of atrial fibrillation was managed after an integrated cardio-oncologic evaluation: anticoagulation was started in 4 (57.1%) patients and beta-blockers or amiodarone in 5 (71.4%). One patient underwent electric cardioversion and another patient pacemaker positioning to normalise heart rate in order to continue ibrutinib.

**Conclusion:**

Our data show that echocardiography is a highly informative and reproducible tool that should be included in pre-treatment workup for patients who are candidates for ibrutinib therapy.

**Electronic supplementary material:**

The online version of this article (10.1186/s13045-018-0626-0) contains supplementary material, which is available to authorized users.

To the Editor,

Ibrutinib is an oral inhibitor of Bruton’s tyrosine kinase indicated for chronic lymphocytic leukaemia (CLL) treatment [1]. Most common side effects are bleeding and ibrutinib-related atrial fibrillation (IRAF) [2, 3]. IRAF pathogenesis is still unclear, and a direct drug effect on myocardiocyte signalling has been postulated [4]. Various *real-life* cohorts have been retrospectively reported, focusing on ibrutinib-related adverse event management [5, 6]. A retrospective study on a large population of CLL patients revealed an association of AF risk with older age, male sex, valvular heart disease, and hypertension [7]. However, no prospective ad hoc studies are available. We systematically analysed predictors of IRAF in 43 CLL patients treated with ibrutinib, focusing on comorbidities, electrocardiographic features (12-derivation electrocardiography (ECG), 24-h ECG monitoring in selected case), and trans-thoracic echocardiography (TTE). Framingham Heart Study and Shanafelt risk score for AF were also calculated for each patient (Additional file 1: Supplementary material) [7, 8].

Patients’ clinical and biological characteristics together with baseline cardiologic comorbidities are presented in Table 1 (Additional file 1: Supplementary results). Previous paroxysmal AF was reported in 5 patients, but in none of them was detectable at the time of cardiologic evaluation nor in the previous 6 months. AF-risk score individuated 8 patients at low, 18 at intermediate low, 6 at intermediate high, and 11 at high risk. Twenty-three patients were taking cardio-active therapy, 13 were under antiplatelets, and 4 under anticoagulant therapy at the time of enrolment. ECG and ECG-Holter analysis did not display any remarkable alterations (Additional file 1: Table S1). TTE demonstrated left atrial (LA) diameter and area over the 75° percentile in 16 and 30% of patients, respectively, although with preserved function. Median observation period was of 8 months (range 1–37). AF occurred in 7 patients (16.3%) after a median of 6 months (range 1–20) from the beginning of ibrutinib. In 5 patients (71%), IRAF developed within 6 months, whereas it occurred within the first year in the other two. AF severity was G2 in 4 patients, G3 in 2, and G1 in 1 only. IRAF cases were all elderly pre-treated males in advanced stage, and one of them had previous history of AF and 6 (85.7%) had arterial hypertension. IRAF incidence significantly correlated with male gender (*p* = 0.04) and history of arterial hypertension (*p* = 0.009). On the whole, IRAF occurred in 25% of the 28 patients with one or more pre-existent cardiologic comorbidities, whereas incidence was 0 in those without (*N* = 15, *p* = 0.03; Additional file 1: Table S2). Of note, a high AF-risk score was detected in 86% of patients who developed IRAF versus 14% in those who did not (*p* < 0.001, Fig. 1, upper panel). No correlation was found with basal ECG evaluation, whereas TTE data showed a significant association with IRAF incidence; IRAF cases displayed higher LA diameter (*p* = 0.02) and area (*p* = 0.03; Fig. 1, lower panel) compared to the others. The occurrence of AF was managed with anticoagulation in four (57.1%) patients, and anti-arrhythmic drugs in five (71.4%). Only one patient underwent electric cardioversion. As regards new onset of arterial hypertension, we observed four events (3 grade III, 1 grade II) after a median of 4.8 months (range 1–27). All four cases were managed with therapy modification, without ibrutinib discontinuation.

**Table 1:** Baseline characteristics

		*N* (%)
	Age years	72 (51–86)
	Gender	F14 (32) M29 (68)
	Follow-up months	8 (1–37)
STAGE	Rai I/II	23 (53)
	Rai III/IV	20 (47)
	Binet A/B	26 (60)
	Binet C	17 (40)
FISH	del13	13 (30)
	trisomy 12	3 (7)
	del17p or TP53 mut	12 (28)
	del11q	5 (12)
VHIG	Mutated	10/36 (28)
	Unmutated	26/36 (72)
Treatment	First-line	12 (28)
	≥Second line	31 (72)
Cardiovascular comorbidities	Previous AF	5 (12)
	AH	23 (53)
	Valvular heart disease	20 (47)
	CAD	8 (19)
	PAD	4 (9)
	Diabetes	4 (9)
	Hypothyroidism	5 (12)
	Dislipidaemia	12 (28)
	Smoke	1 (2)
	BMI > 25	7 (16)

Values are given as median (range) or as *N* (%). *AH* arterial hypertension, *CAD* coronary artery disease, *PAD* peripheral artery disease, *BMI* body mass index

**Fig. 1:**
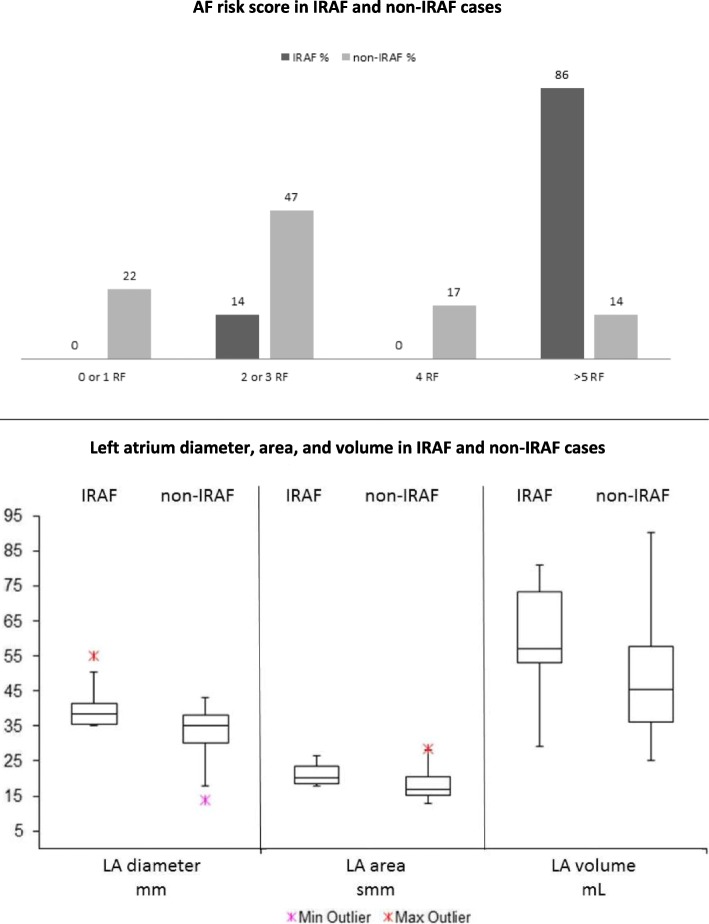
Upper panel: AF risk score according to IRAF incidence. AF risk score was calculated on age, gender, arterial hypertension, and valvular heart disease (Shanafelt et al.). Percentages of patients developing or not developing IRAF are shown divided into four risk categories. IRAF cases were mainly in the high risk group (≥ 5 RF), *p* < 0.001. *AF* atrial fibrillation. *IRAF* ibrutinib-related atrial fibrillation. *RF* risk factors. Lower panel: main echocardiographic characteristics associated with IRAF. LA diameter, area, and volume were increased in IRAF patients compared to the others, significantly for the former two parameters (*p* = 0.02 and *p* = 0.03, respectively). *LA* left atrium, *IRAF* ibrutinib-related atrial fibrillation

In this prospective analysis, we firstly show that baseline echocardiographic characteristics significantly predict IRAF occurrence. We describe a higher incidence of IRAF compared to previous reports, possibly due to closer cardiologic evaluation. Accordingly, methods for IRAF screening were not detailed in previous studies, with possible underestimation [8]. In the Shanafelt series, only 72 out of 2444 patients were treated with ibrutinib and only 2 of them developed IRAF. As a matter of fact, this score is more predictive of AF in CLL general population than of IRAF. However, Shanafelt risk score significantly predicted IRAF development in our study, indicating high reproducibility. As regards echocardiography, in our series, TTE-measured LA linear dimensions and area clearly correlated with higher IRAF incidence, pointing out that it may be a useful and non-invasive tool to assess IRAF risk in ibrutinib candidates. Finally, similarly to previous experiences, only half of IRAF cases received anticoagulation because of both high bleeding risk and possible drug interactions [5, 7, 9]. In conclusion, TTE is a highly reproducible, widespread, and low-cost procedure that might be easily included in the pre-treatment workup of ibrutinib candidates, and self-interpreted by a skilled haematologist.

## Additional file

Additional file 1: Supplementary material and results. (DOC 60 kb)

## Abbreviations

BMI: Body mass index
CLL: Chronic lymphocytic leukaemia
ECG: Electrocardiography
IRAF: Ibrutinib-related atrial fibrillation
LA: Left atrium
TTE: Trans-thoracic echocardiography
