# Supplementary material for: Predictors of atrial fibrillation in ibrutinib-treated CLL patients: a prospective study
Source: J Hematol Oncol. 2018 Jun 11;11:79. doi: 10.1186/s13045-018-0626-0 (PMC5996546; doi:10.1186/s13045-018-0626-0)
Supplement: Supplementary file 1 — Supplementary material and results. (DOC 60 kb) [file 13045_2018_626_MOESM1_ESM.doc]

**Supplementary materials**

We enrolled chronic lymphocytic leukemia (CLL) patients eligible for ibrutinib treatment according to current guidelines (Eichhorst B, et al. Ann Oncol 2015; Hallek M, et al. Blood 2008), from June 2014 to June 2017, at our Institution. Age and gender, clinical and biological information, including the date of diagnosis, Rai/Binet staging system, treatment history, FISH and IGHV analysis were collected.

Before starting therapy each patient underwent a full cardiologic evaluation by an expert cardio-oncologist. Clinical history and physical examination were performed at enrolment and along the follow up, focusing on cardiovascular risk factors assessment. Comorbidities were also investigated including arterial hypertension, diabetes mellitus, chronic valvular heart disease, dislipidemia, smoking history, ischemic coronaropathy and peripheral arteriopathy. Chronic pulmonary disease was excluded in each patient. Height and weight were used to calculate the body mass index (BMI). The use of concomitant cardioactive drugs at the time of Ibrutinib initiation was assessed. All CLL patients underwent baseline non-invasive instrumental investigations: 12-derivations electrocardiography (ECG) was used to assess sinus rhythm, intra-atrial and atrioventricular abnormal conduction. Trans-Thoracic Echocardiogram (TTE) was performed to measure global systolic and diastolic function, left atrium (LA) size and function including linear dimensions (E/A and diameter), LA area and volume were also calculated.

A 24 hours ECG monitoring was performed for further heart rhythm evaluation. Finally, the Framingham Heart Study AF risk was calculated for each patient (Schnabel RB, et al. Lancet 2009). The predictive risk score for developing AF, designed by Shanafelt et al., was also calculated (Shanafelt TD, et al. Leuk Lymphoma 2017). Each patient was educated about symptoms related to AF, and general cardiac evaluation was performed by the care-giver hematologist at least monthly for early asymptomatic AF detection. AF severity was also evaluated according to Common Terminology Criteria for Adverse Events (CTCAE) Version 4.0 (June14, 2010).

For statistical analysis, demographic variables were reported using descriptive statistics. Means were compared using the Student’s t-test. The Chi-squared or Fisher’s exact tests were used for the comparison of categorical variables, where appropriate.

**Supplementary results**

**Patients’ baseline characteristics**: male/female ratio was 2.07 and median age 72 years. Median time from diagnosis to ibrutinib therapy was 7.9 years (range 1.71-17.9 years). Considering Rai/Binet stages, 47% of patients displayed Rai III/IV and 40% Binet C at baseline. Concerning biologic prognostic factors, 26 patients presented with at least one FISH abnormal finding, and 7 with two combined alterations. Adverse prognostic FISH abnormalities (del11q and del17p/TP53 mutation) were found in 39.5% of cases. Finally variable heavy chain Ig status was unmutated in 72% of the 36 patients tested. The majority of patients had been previously treated with at least one therapy line (median 2, range 1-6), mostly chemo-immunotherapy (11 with fludarabine-cyclophosphamide-rituximab, FCR; 9 with bendamustine with rituximab or ofatumumab; 5 with alemtuzumab; and 7 with chlorambucil).

**Baseline cardiologic comorbidities**: 28 patients (65%) had at least one cardiologic comorbidity, mostly arterial hypertension (53.5%) and mild valvular heart disease (47%). Previous paroxysmal AF was reported in 5 patients, but in none of them was detectable at the time of cardio-oncologist evaluation, nor in the previous 6 months. Moreover, 1 patient had a smoking history and 2 were overweight (BMI>25, median values for all cases 23.55, 16.8-30.3). Atrial fibrillation risk score individuated 8 patients at low, 18 at intermediate-low, 6 at intermediate-high, and 11 at high risk (reported risk of AF from 4 to 33%). Finally, moderate or severe chronic respiratory diseases (in particular chronic obstructive disease, COPD) were excluded in all patients. 23 patients were taking chronic cardio-active therapy: calcium channel blockers in 3, beta-blockers in 12, diuretics in 7, ACE-inhibitors in 10, and flecainide and amiodarone antiarrhythmic drugs in 2 cases. Moreover, 13 patients were under antiplatelets and 4 under anticoagulant therapy.

**Echo- and electro-cardiographic data at baseline**: ECG analysis did not display any remarkable alterations in heart rate (HR), rhythm, atrial conduction, atrial-ventricular conduction, nor repolarization. TTE demonstrated normal diastolic and systolic function, and no clinically relevant valvular abnormalities in all cases. However, 16% of cases presented with LA diameter and 30% with LA area over the 75^o^ percentile (diameter > 40 mm and area > 20 smm, respectively), although with preserved function. ECG Holter evaluation showed median maximal HR of 91 ppm (88-119), median minimal HR of 52 ppm (42-62), median number of supra ventricular ectopic beats (SVEB) of 23.5/24h (0-130), and I grade atrial ventricular block (AVB) in 4 cases.

**Predictors of IRAF**: IRAF cases were all elderly males; all but one of them presented with Rai stage III/IV and had been already treated with chemo-immunotherapy. All IRAF patients displayed FISH abnormalities, in particular two del11q and three del17p/TP53 mutation. Finally, 3 cases had unmutated variable heavy chain Ig status. Among IRAF patients only one (14.2%) had previous history of AF and 6 (85.7%) had arterial hypertension. We found a significant correlation between IRAF incidence and male gender (p=0.04), as well as with history of previous arterial hypertension (p=0.009). On the whole, IRAF occurred in 25% of the 28 patients with one or more pre-existent cardiologic comorbidities, whereas the incidence was 0 in those without (15 patients, p=0.03).

No significant correlation emerged between concomitant cardio-active therapy and IRAF occurrence. Of note, AF risk score was high in 86% of patients who developed IRAF versus 14% in those who did not (p<0.001, Figure 1). Basal ECG evaluation displayed no correlation with IRAF occurrence. At variance, TTE data showed a significant association with IRAF incidence. IRAF cases displayed higher LA diameter (p=0.02) and area (p=0.03). Moreover, mean ejection fraction was slightly lower in IRAF cases. Finally, concerning prolonged ECG-monitoring, patients who developed IRAF showed a higher maximal heart rate (HR) compared to those in stable sinus rhythm (table 4), although not significantly.

**IRAF management**: all events were managed after an integrated cardio-oncological evaluation: anticoagulation was started in 4 (57.1%) patients, and beta-blockers or amiodarone in 5 (71,4%). One patient underwent electric cardioversion and another patient pace maker positioning to normalize heart rate in order to continue ibrutinib.

**Arterial hypertension management**: during the study period we observed 4 events; 3 patients introduced diuretics, 2 alpha-lytic drugs, 2 calcium channel blockers, and 1 ACE-inhibitor, with prompt clinical control and no ibrutinib discontinuation. Finally, 3 cases displayed minor bleedings, all resolved after topical therapy.

| **Table S1. echo- and electro-cardiographic evaluation** | |
| --- | --- |
|  |  |
| **EF %** | 65 (60-70) |
| **MR N(%)** | 18 (42) |
| **E/A** | 0.77 (0.4-1.95) |
| **LA diameter mm** | 37 (14-55) |
| **LA volume mmc** | 46.65 (25.3-104.2) |
| **LA area smm** | 17.75 (13-28.4) |
| **QT msec** | 0.4(0.16-0.48) |
| **PQ msec** | 0.18 (0.08-0.48) |
| **HR ppm** | 66 (42-110) |
| **mean HR ppm** | 69 (52-89) |

Values are given as median (range) or number(%).MR mitral regurgitation. HR heart rate. EF ejection fraction. MR mitral valve regurgitation. LA left atrium.

| **Table S2. Baseline characteristics predictive of IRAF development.** | | |
| --- | --- | --- |
|  | **IRAF N=7** | **Non-IRAF N=35** |
| **Age** years | 77+2,7 | 71+9,8 |
| **Male gender** | 7 (100) | 21 (60)* |
| **QT** msec | 0,38+0,1 | 0,38+0,06 |
| **PQ** msec | 0,22+0,15 | 0,19+0,04 |
| **HR** ppm | 62,2+13,7 | 70,3+15,5 |
| **Mean HR** ppm | 72,5+3,4 | 69,3+13,5 |
| **24hours electrocardiography** | | |
| **HR** max | 109,5+0,7 | 73,35+30,6** |
| **HR** min | 50+0 | 76,78+37,9 |
| **SVEB** | 16+22,6 | 35,2+39,4 |
| **pause/AVB** | 0 | 0,07+0,26 |
| **Echocardiography** | | |
| **EF** | 61,7+4 | 64,5+4,3** |
| **MR N(%)** | 4 (57) | 16 (46) |
| **E/A** | 0,53+0,14 | 0,91+0,36* |
| **LA diameter** mm | 40,6+7,5 | 34,2+7,01* |
| **LA volume** mL | 59+18,9 | 49,06+17,7 |
| **LA area** smm | 21,25+3,5 | 18,1+3,7* |

Values are given as mean+standard deviation or number(%). IRAF ibrutinib associated atrial fibrillation. HR heart rate. SVEB supraventricular ectopic beats. AVB atrial-ventricular block. EF ejection fraction. MR mitral valve regurgitation. LA left atrium. *<0.05; **=0.07
